# Dual EGFR inhibition in combination with anti-VEGF treatment: A phase I clinical trial in non-small cell lung cancer

**DOI:** 10.18632/oncotarget.763

**Published:** 2013-01-14

**Authors:** Gerald S. Falchook, Aung Naing, David S. Hong, Ralph Zinner, Siqing Fu, Sarina A. Piha-Paul, Apostolia M. Tsimberidou, Sonia K. Morgan-Linnell, Yunfang Jiang, Christel Bastida, Jennifer J. Wheler, Razelle Kurzrock

**Affiliations:** ^1^ Department of Investigational Cancer Therapeutics (Phase I Program), U.T. MD Anderson Cancer Center, Houston, TX; ^2^ Moores Cancer Center at the University of California, San Diego

**Keywords:** Erlotinib, Cetuximab, Bevacizumab, EGFR, VEGF

## Abstract

**BACKGROUND:**

Preclinical data indicate EGFR signals through both kinase-dependent and independent pathways and that combining a small-molecule EGFR inhibitor, EGFR antibody, and/or anti-angiogenic agent is synergistic in animal models.

**METHODS:**

We conducted a dose-escalation, phase I study combining erlotinib, cetuximab, and bevacizumab. The subset of patients with non-small cell lung cancer (NSCLC) was analyzed for safety and response.

**RESULTS:**

Thirty-four patients with NSCLC (median four prior therapies) received treatment on a range of dose levels. The most common treatment-related grade &ge;2 adverse events were rash (n=14, 41%), hypomagnesemia (n=9, 27%), and fatigue (n=5, 15%). Seven patients (21%) achieved stable disease (SD) &ge;6 months, two achieved a partial response (PR) (6%), and two achieved an unconfirmed partial response (uPR) (6%) (total=32%). We observed SD&ge;6 months/PR/uPR in patients who had received prior erlotinib and/or bevacizumab, those with brain metastases, smokers, and patients treated at lower dose levels. Five of 16 patients (31%) with wild-type EGFR experienced SD&ge;6 months or uPR. Correlation between grade of rash and rate of SD&ge;6 months/PR was observed (p<0.01).

**CONCLUSION:**

The combination of erlotinib, cetuximab, and bevacizumab was well-tolerated and demonstrated antitumor activity in heavily pretreated patients with NSCLC.

## INTRODUCTION

The epidermal growth factor receptor (EGFR) is a receptor tyrosine kinase that plays an important role in tumorigenesis [1], and signals via downstream effectors [2]. EGFR mutations are seen in ~13% of patients with non-small cell lung cancer (NSCLC) in the United States [3], with a higher incidence in Japanese patients [4], and contribute to the pathogenesis of affected lung tumors. Targeted therapies have shown promise in the treatment of NSCLC, with studies selecting patients for molecular targets faring better in general [5-8]. Erlotinib, an EGFR inhibitor, is approved by the Federal Drug Administration (FDA) to treat locally advanced or metastatic NSCLC [9]. Most responses to erlotinib occur in patients with EGFR mutations [3, 10-12], and both resistant (e.g., L861Q) and sensitive (e.g., exon 18 G719S, exon 19 deletion or exon 21 L858R point mutation) mutations have been identified [12-15]. Cetuximab, a monoclonal antibody to EGFR, has demonstrated efficacy in NSCLC when combined with chemotherapy [16], but is not currently FDA-approved for NSCLC.

Recently, Weihua, et al. [17] discovered that EGFR can maintain cancer cell survival independent of its kinase activity. This kinase-independent pathway operates via increased glucose uptake due to stabilization of the SGLT1 glucose transporter, with a downstream effect of reduced autophagy [17]. Therefore, targeting both kinase-dependent and kinase-independent EGFR functions may be a rational treatment strategy. Indeed, studies in animal models revealed that combining antibodies and kinase inhibitors was synergistic [18, 19]. Cetuximab blocks receptor activation by interfering with ligand binding, as well as down-regulating EGFR levels and inhibiting cell growth in association with inhibition of ligand-independent EGFR signaling [20, 21]. Therefore, it is plausible that treatment with cetuximab could suppress kinase-independent cell signaling [21].

Angiogenesis also plays an important role in tumor development and metastasis [22], mediated in large part by vascular endothelial growth factor (VEGF) and its receptor VEGFR [23]. Bevacizumab is a recombinant anti-VEGF monoclonal antibody FDA-approved for treatment of unresectable, locally-advanced, recurrent, or metastatic NSCLC in combination with paclitaxel and carboplatin [9, 24].

Unfortunately, targeting angiogenesis or EGFR alone does not provide adequate tumor control in many patients [25-27]. Prior studies combining erlotinib and cetuximab or gefitinib and cetuximab failed to demonstrate tumor regressions in patients with lung adenocarcinomas resistant to erlotinib [28] or patients with NSCLC previously treated with platinum-based therapy [29], respectively. In contrast, targeting both VEGF and EGFR pathways demonstrated synergy in vivo [30, 31], possibly because resistance to EGFR inhibitors may be mediated at least partly by activating VEGF-dependent signaling as an alternative survival pathway [30, 31]. Furthermore, combining erlotinib and bevacizumab in patients with NSCLC who had not received prior anti-VEGF or anti-EGFR treatment, showed response rates (CR/PR) of 18-20% [31, 32] and improved progression-free survival (PFS), but not overall survival (OS) [33].

Here, we report, for the first time, the results of administering dual EGFR inhibitors (erlotinib plus cetuximab) together with an anti-angiogenic agent (bevacizumab) in 34 patients with heavily-pretreated NSCLC.

## RESULTS

### Demographics

Thirty-four patients with NSCLC were enrolled (Table 2). All patients had progressive disease at the time of enrollment. Most patients were heavily pretreated, with a median of four prior therapies (range 1-8). Most patients (76%) had adenocarcinoma histology. Thirteen patients (38%) were previously treated with erlotinib, and 11 patients (32%) had previously received bevacizumab. Three of 19 patients tested (16%) had EGFR mutations; 19 patients tested for KRAS mutations were all wild-type. The only patient tested for p53 had a mutation (R196*), and two patients out of nine tested had PIK3CA mutations (E542K and E545K). PTEN expression was tested by immunohistochemistry (n=3) or mutation analysis (n=2), and no aberrations were found.

### Adverse Events

The most common treatment-related grade 2 or higher adverse events were rash (n=14, 41%), hypomagnesemia (n=9, 26%), and fatigue (n=5, 15%) (Table 1). Treatment-related grade 2 hypotension, hemoptysis, neuropathy, vomiting, anorexia, infusion reaction, proteinuria, and grade 3 diarrhea were each observed in only one patient. Two patients experienced dose-limiting toxicity (DLT), including grade 3 rash (n=1, dose level 8) and grade 3 bronchospasm (n=1, dose level 6). Twenty-four patients (71%) experienced no drug-related toxicity higher than grade 1. Three patients (9%) withdrew due to toxicity, including grade 3 bronchospasm in cycle 1 (n=1), grade 1 diarrhea in cycle 1 (n=1), and grade 3 fatigue in cycle 4 (n=1). No deaths resulted from adverse events. The RP2D was level 8, which includes the recommended FDA-approved full dose of each medication [34].

**Table 1:** Treatment-related Grade 2-4 adverse events observed in ≥ 5% of patients

	Dose Level	1 n=1	2 n=1	3 n=5	4 n=2	5 n=2	6 n=6	7 n=3	8† n=14	Total n=34
	Bevacizumab Dose, mg/kg IV q2w	2.5	5.0	5.0	5.0	7.5	7.5	7.5	10.0	
	Cetuximab Dose, mg/m2 IV weekly	100, 75*	100, 75	200, 125	200, 125	200, 125	400, 250	400, 250	400, 250	
	Erlotinib Dose, mg PO daily	50	50	50	100	100	100	150	150	
Fatigue										
Grade 2		0	0	0	0	0	2	0	2	4 (12%)
Grade 3-4		0	0	0	0	0	0	0	1	1 (3%)
Rash^a^										
Grade 2		0	0	3	1	0	1	1	5	11 (32%)
Grade 3-4		0	0	0	0	0	0	0	3 (DLT)^b^	3 (9%)
Hypomagnesemia									
Grade 2		0	1	0	1	0	0	0	1	3 (9%)
Grade 3-4		0	0	0	1	0	1	0	4	6 (18%)
Nausea										
Grade 2		0	0	0	0	0	1	0	1	2 (6%)
Grade 3-4		0	0	0	0	0	0	0	0	

Abbreviations: DLT, dose-limiting toxicity; IV, intravenous; po, orally; q2w, every 2 weeks

† Recommended Phase II dose[33] (full approved doses of each drug).

* Cetuximab dose shown as loading dose, maintenance dose.

a Including pruritis

b One out of the three events was considered a DLT.

**Table 2:** Patient Demographics

Characteristics (n=34)	
Age (years)	
Median	62
Range	27-78
Gender, n (%)	
Men	18 (53%)
Women	16 (47%)
Histologies, n (%)	
Adenocarcinoma	26 (76%)
Squamous cell	3 (9%)
Mucinous adenocarcinoma	2 (6%)
Poorly differentiated carcinoma	2 (6%)
Sarcomatoid carcinoma	1 (3%)
No. of prior systemic therapies, n (%)	
Median	4
Range	1-8
Prior bevacizumab, n (%)	11 (32%)
Prior EGFR inhibitors, n (%)	13 (38%)
EGFR mutations, n (%)	
Positive	3 (9%)
Negative	16 (47%)
Unknown	15 (44%)
KRAS mutations, n (%)	
Positive	0 (0%)
Negative	19 (56%)
Unknown	15 (44%)
History of smoking	23 (68%)
History of brain metastases	11 (32%)
ECOG performance status score, n (%)	
0	2 (6%)
1	31 (91%)
2	1 (3%)

Abbreviations: ECOG, Eastern Cooperative Oncology Group

### Responses

**Figure 1:**
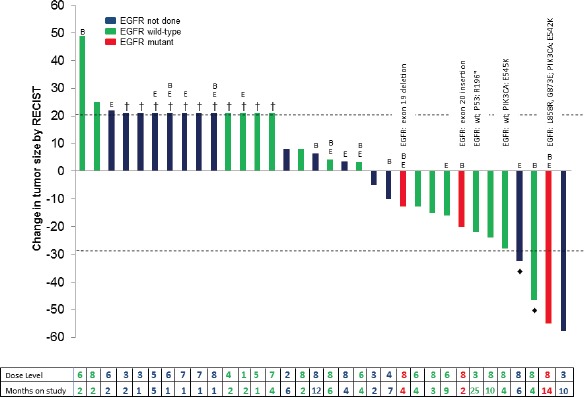
Best response in all 34 NSCLC treated Patients with early clinical progression, new lesions, or who withdrew early are indicated arbitrarily as +21% and denoted by †. Unconfirmed PRs are indicated by ♦. Patients who received prior bevacizumab are denoted by “B”, and patients who received prior erlotinib are denoted by “E”. No patients had cetuximab. Patients with wild-type EGFR are shown in green; patients with EGFR alterations are shown in red; and patients that were not tested for EGFR mutations are shown in blue. The specific mutation in EGFR, PIK3CA, and/or p53 is labeled for all patients that had mutations in one or more genes tested. The dose level and treatment duration (months) for each patient are shown in the table below.

**Figure 2:**
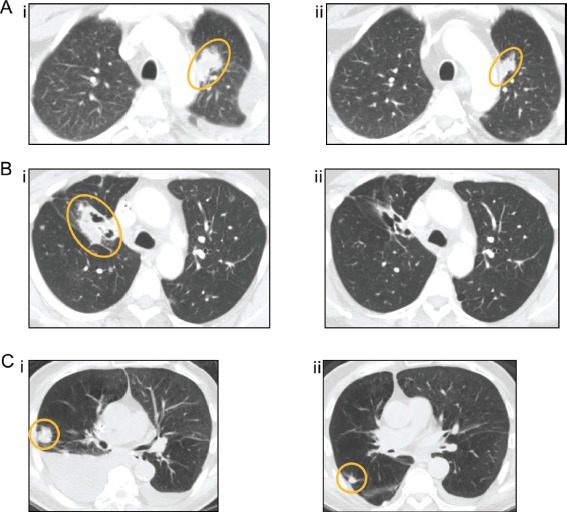
Computed tomography (CT) images of the three patients with the greatest tumor reduction (A) patient #37 (EGFR mutation not done, smoker), who achieved a PR (59% decrease), at baseline (i) and 32 weeks (ii), (B) patient #197 (EGFR L858R, G873E and PIK3CA E542K mutations, nonsmoker) who achieved a PR (55% decrease), at baseline (i) and 20 weeks (ii), and (C) patient #200 (EGFR wild-type, smoker), who achieved an unconfirmed PR (48% decrease), at baseline (i) and 8 weeks (ii).

All 34 patients are included in the response data (Figure 1). Three patients withdrew before the first restaging assessment due to toxicity, and one patient withdrew early because of financial considerations. These four patients and any patients with clinical progression or new lesions are arbitrarily depicted as 21% increase (Figure 1) and are considered treatment failures. Four patients (12%) achieved a PR (two were unconfirmed PR (uPR)) and received treatment for 4, 6, 10, and 14 months (Table 3, Figure 2). Seven patients (21%) achieved stable disease (SD) lasting at least 6 months (duration was 6, 6, 7, 9, 10, 12, and 25 months) (total SD≥6 months/PR/uPR=11, (32%)).

**Table 3:** Characteristics for patients with any tumor regression or SD≥6 months (n=17)

Case #	Histology	Best Response %	Months on study	Smoker	EGFR	PIK3CA	p53	Prior EGFR Tx	Prior bevacizumab	Brain metastases	Dose Level	Rash Grade >3
PR												
37	Adenocarcinoma	−59	10	Y	ND	ND	ND	N	N	Y	3	N
197	Mod. diff. adeno.	−55	14	N	L858R G873E	E542K	ND	Erlotinib (13 months)	Y	Y	8	N
200	Poorly-mod. diff. adeno.	−48♦	4	Y	NEG	NEG	ND	N	Y	Y	8	Y
226	Adenocarcinoma	−34♦	6	N	ND	ND	ND	Erlotinib (10 months)	N	N	8	N
SD≥6 Months												
153	Poorly diff. adeno. with mucin	−24	10	N	NEG	NEG	ND	N	N	N	8	Y
39	SCC	−22	25	Y	NEG	NEG	R196*	N	N	N	3	N
90	Mucinous adeno	−16	9	N	NEG	NEG	ND	Erlotinib (5 months)	N	N	6	N
45	Adenocarcinoma	−10	7	Y	ND	ND	ND	N	Y	Y	4	N
146	Adenocarcinoma	5	6	Y	NEG	NEG	ND	Erlotinib (2 months)	Y	Y	8	N
169	Adenocarcinoma	7	12	Y	ND	ND	ND	N	Y	Y	8	N
28	Adenocarcinoma	8	6	Y	ND	ND	ND	N	N	N	2	N
SD<6 Months and tumor decrease 0-29%												
228	Adenocarcinoma	−28	4	Y	NEG	E545K	ND	N	N	N	8	N
148	Adenocarcinoma	−20	2	N	Exon 20 insertion	ND	ND	N	Y	N	8	Y
207	Adenocarcinoma	−15	3	Y	NEG	NEG	ND	N	N	N	8	N
89	Poorly diff. adeno.	−13	4	Y	NEG	ND	ND	N	N	N	6	N
181	Adenocarcinoma	−11	4	Y	Exon 19 deletion	ND	ND	Erlotinib (12 months)	Y	N	8	N
40	Adenocarcinoma	−5	2	Y	ND	ND	ND	N	N	Y	3	N

Abbreviations: adeno, adenocarcinoma; diff, differentiated; mod, moderately; ND, not done; NEG, negative; PR, partial response; SCC, squamous cell carcinoma; SD; stable disease; Tx, treatment

♦ Indicates an unconfirmed PR.

### Prior EGFR inhibitor or VEGF Inhibitor Therapy and Response

Of all 34 patients on study, seven patients (21%) had received prior erlotinib but no prior bevacizumab, five patients (15%) had received prior bevacizumab but no prior erlotinib, and six additional patients (18%) had received prior erlotinib and bevacizumab (five patients received prior sequential erlotinib and bevacizumab; one patient received prior concurrent erlotinib and bevacizumab). No patients had previously received cetuximab. Of seven patients who received prior erlotinib but no prior bevacizumab, two (29%) had SD≥6 months/uPR. Of the five patients who received prior bevacizumab but no prior erlotinib, three (60%) had SD≥6 months/uPR. Of the six patients who had received both prior erlotinib and bevacizumab, two (33%) achieved SD≥6 months/PR. The patient who received prior concurrent erlotinib and bevacizumab did not achieve SD≥6 months/PR.

Of the 11 patients with SD≥6 months/PR/uPR, four (36%) had prior EGFR inhibitor treatment (two with erlotinib alone and two with prior sequential erlotinib and bevacizumab), five (45%) had prior bevacizumab (three with bevacizumab alone and two with prior sequential erlotinib and bevacizumab), and four (36%) had neither prior erlotinib or bevacizumab (Table 3). Prior EGFR and/or antiangiogenic treatment did not preclude SD≥6 months/PR/uPR.

Among the four patients with SD≥6 months/PR/uPR who had received prior erlotinib, one patient had primary resistance to erlotinib alone, having developed progression after two months of erlotinib (Table 3, patient #146). Three patients (75%) received the combination treatment for as long or longer than the duration of the prior erlotinib (Table 3, patients #197, 90, and 146) (2, 5, and 13 months, respectively, with initial erlotinib treatment, and 6, 9, and 14 months, respectively, with the combination treatment). Overcoming primary resistance to erlotinib and achieving a longer duration of treatment with this combination was demonstrated.

### Brain Metastases and Response

Of 11 patients with brain metastases, three achieved a PR/uPR, and three had SD≥6 months (total six of 11, 54%) (Table 3). One of these patients had five small, untreated brain metastases and achieved complete resolution of the brain tumors. The presence of brain metastases did not preclude SD≥6 months/PR/uPR.

### Smoking History and Response

Twenty-three (68%) of the patients had a history of smoking. Of these 23 patients, two (9%) achieved a PR/uPR, and five (22%) had stable disease SD≥6 months (total=31%), including one patient who was treated for 25 months (Table 3). Of the 11 patients (32%) that did not have a history of smoking, two achieved a PR/uPR, and two had SD≥6 months (total=36%) (Table 3). Smoking history did not preclude SD≥6 months/PR/uPR.

### Dosing and Response

Of 17 patients on dose levels 7 or 8, six (35%) achieved SD≥6 months/PR/uPR. For patients treated at dose levels 1-6, five of 17 (29%) achieved SD≥6 months/PR (Table 1 and Figure 1). The only patient treated on dose level 2 was stable for 6 months; two of the five patients treated on dose level 3 achieved SD≥6 months/PR. There was no obvious dose-response correlation, although the number of patients was small.

### Molecular Aberrations and Responses

Of 16 patients with documented wild-type EGFR, five (31%) had SD≥6 months or uPR. Three of 19 patients tested for EGFR aberrations showed an abnormality, and one of the three patients, with an activating L858R mutation [35] and a G873E mutation, had a PR. This patient also demonstrated a co-existing PIK3CA mutation and had received prior sequential erlotinib and bevacizumab; on the current study, the patient has achieved a 55% regression and received treatment for 14 months. The other two patients with EGFR aberrations (exon 20 insertion and exon 19 deletion) demonstrated 20% and 11% regression, respectively, as best response, but the regressions were short—2 and 4 months on treatment, respectively. The presence of wild-type EGFR did not preclude achieving SD≥6 months/uPR.

The only patient tested for p53 mutations had an R196* nonsense mutation. This patient achieved a 22% regression lasting 25 months.

Two of the nine total patients tested demonstrated PIK3CA mutations. One had an E542K mutation (with concomitant EGFR mutation) and had a 55% regression, as mentioned above. The other patient, with an E545K mutation, had a 28% decrease (treatment duration=4 months). The presence of PIK3CA mutations did not preclude response.

Three patients were tested for PTEN loss, and in all patients, PTEN expression was normal. While PTEN loss can reflect a genetic aberration in NSCLC [36], the two patients tested for PTEN mutation were wild-type.

### Toxicity and Response

Rash was the most frequently observed toxicity in patients (Table 1); 11 patients experienced grade 2 rash, of whom five (45%) achieved SD≥6 months/PR/uPR. Two of three patients with grade 3 rash or higher achieved SD≥6 months/uPR (Table 3). Of 20 patients with grade 1 or no rash, four (20%) achieved SD≥6 months/PR/uPR. Patients with higher grade rash were significantly more likely to have SD≥6 months/PR/uPR (Spearman's correlation coefficient, p<0.01).

## DISCUSSION

In this study, we report the results of the cohort of patients with NSCLC treated on a phase I dose-escalation trial of combination bevacizumab, cetuximab, and erlotinib. The rationale for this combination was: (1) preclinical and clinical studies that suggested increased activity when erlotinib was combined with bevacizumab [30, 31]; (2) preclinical studies indicating that EGFR signals through both kinase-dependent and -independent pathways [17]; and (3) studies demonstrating that combining an EGFR kinase inhibitor with EGFR antibodies was synergistic in animal models [18, 19].

This combination of drugs was well-tolerated. The RP2D was determined to be the full FDA-approved doses for all three drugs [34], and 13 of the 14 patients (93%) treated at the RP2D tolerated treatment without drug-related dose-limiting effects.

This regimen demonstrated antitumor activity in patients with NSCLC, including 11 patients (32%) who had a best overall response of SD≥6 months (n=7) or PR (n=4) (two PRs were unconfirmed). SD≥6 months/PR/uPR were observed even in patients who had received prior bevacizumab and/or erlotinib, those with brain metastases, smokers, and patients treated at lower dose levels, so these characteristics do not preclude antitumor activity.

Remarkably, patients who previously had failed erlotinib also achieved SD≥6 months/PR/uPR. In fact, overcoming primary resistance to erlotinib and achieving a longer duration of treatment with this combination than with prior erlotinib alone was demonstrated. Recent preclinical studies suggest that combining EGFR kinase inhibitors and anti-EGFR antibodies may be more effective than either alone, perhaps because EGFR is able to maintain cancer cell survival independent of its kinase activity [17-19]. The clinical data presented here also support combining kinase inhibitors and antibodies.

Previous phase I/II clinical studies combining cetuximab and erlotinib in patients with NSCLC failed to show significant tumor regression [28]. In contrast, as mentioned above, four patients on our study achieved a PR/uPR. The reason for this difference is unclear but could be due to the addition of bevacizumab in our regimen or because almost half of the patients treated on the prior study (but none in our study) had a known EGFR resistance mutation [37, 38]. It is important to use caution comparing the previous study to the results presented here because the previous study was conducted in a select group of patients, i.e., only patients with NSCLC who had received erlotinib throughout one month prior to enrollment and who had clinically-defined erlotinib resistance.

Prior studies combining erlotinib and bevacizumab showed PR/CR rates of 18-20% and improved PFS, but no improvement in OS, supporting a potential role for bevacizumab [32, 33]. However, these results cannot be compared directly with those in our study because patients in the prior studies were less heavily pretreated (median 1-2 prior systemic regimens versus four in our study) and both prior studies excluded patients who had received previous EGFR or VEGF inhibitors.

The presence of brain metastases did not compromise the rate of SD≥6 months/PR/uPR. Five of 11 patients with brain metastases achieved SD≥6 months/PR/uPR, and one individual showed complete resolution of her brain metastases. No patient had intracranial hemorrhage. These results suggest that patients with NSCLC and brain metastases can safely receive this regimen and that it has activity, which is consistent with a previous studies on the efficacy of TKIs against brain metastases in NSCLC [39].

SD≥6 months/PR/uPR were observed even at low dose levels. No dose-related difference was observed in the number of patients who achieved SD≥6 months/PR/uPR (six of 17 patients at dose levels 7-8 versus five of 17 patients at dose levels 1-6). These data are consistent with a previous study of 683 patients receiving treatment on phase I trials in our department which found that patients who received lower doses of predominantly targeted agents fared as well as those receiving higher doses [40].

Exploratory analysis of molecular aberrations was performed to identify potential subsets of patients with SD≥6 months/PR/uPR. EGFR, PIK3CA, PTEN, and p53 mutation data were available for 20 patients, including patients who had PTEN assessed by immunohistochemistry. Although the limited number of patients prevents definitive conclusions, it was noted that all five patients with molecular aberrations (EGFR mutations L848R and G873E and PIK3CA mutation E542K (n=1), EGFR exon 20 insertion (n=1), EGFR exon 19 deletion (n=1), p53 mutation (n=1), PIK3CA mutation (n=1)) experienced tumor regression, which in some cases was prolonged.

Of special interest, out of 16 patients with documented wild-type EGFR, five (31%) achieved SD≥6 months/uPR. Although EGFR mutations are generally found in lung cancer patients who are non-smokers [41], seven of 23 smokers (30%) achieved SD≥6 months/PR/uPR. These data suggest that wild-type EGFR and/or a history of smoking did not preclude salutary effects with this regimen.

In regard to toxicity, previous studies have shown a correlation between rash and response to EGFR inhibitors [42]. In our study, patients who had higher grade rash were more likely to have SD≥6 months/PR/uPR (p<0.01). In previous studies of metastatic colorectal cancer combining bevacizumab, cetuximab, and cytotoxic chemotherapy, the addition of cetuximab shortened progression-free survival [43, 44]. In contrast, our trial combined bevacizumab and cetuximab without chemotherapy. Future studies should be pursued to further investigate this combination without chemotherapy.

Despite the promising responses observed in our study, most patients developed progressive disease, which may be explained by multiple potential mechanisms of resistance [45-47]. Future studies should be considered to further investigate causes of resistance in the clinical setting.

In conclusion, the results presented here demonstrate that dual inhibition of EGFR with erlotinib and cetuximab, combined with the VEGF antibody bevacizumab, is well-tolerated, allowing full doses of all three drugs in patients with NSCLC. SD≥6 months/PR/uPR was achieved in 32% of this heavily pretreated patient population, including patients with brain metastases, smokers, those treated at lower doses, those with prior erlotinib and/or bevacizumab, and those with wild-type EGFR. These results suggest that this regimen merits further investigation.

## METHODS

### Study Design

The study was conducted at The University of Texas M. D. Anderson Cancer Center (MDACC) per Institutional Review Board guidelines. The lung cancer cohort reported herein included all patients with NSCLC who started therapy between 4/7/2008 and 12/27/2010 as part of a dose-escalation study conducted in patients with advanced cancer. The dose escalation portion of the study determined the recommended phase II dose (RP2D) to be bevacizumab 10 mg/kg IV every two weeks; cetuximab loading 400 mg/m^2^, maintenance 250 mg/m^2^ IV weekly; and erlotinib 150 mg PO daily [34]. A cycle was 28 days. Patients were treated at variable dose levels, depending on the time of study entry (Table 1).

### Patients

Patients had metastatic or advanced NSCLC not amendable to standard therapy, an Eastern Cooperative Oncology Group (ECOG) performance status 0-2 [48], and adequate hematologic, hepatic, and renal function. Exclusion criteria included hemoptysis, unexplained bleeding, significant cardiovascular disease, intercurrent uncontrolled illness, significant gastrointestinal bleeding within 28 days, hemorrhagic brain metastases, prior abdominal surgery within 30 days, pregnancy, and a history of hypersensitivity to bevacizumab, cetuximab, and/or erlotinib. Treatment with prior cytotoxic therapies must have ended at least three weeks prior to enrollment, and biologic therapy must have ended at least two weeks or five drug half-lives prior to enrollment (whichever is shorter).

### Safety

Clinically significant adverse events were assessed according to the National Cancer Institute Common Terminology Criteria for Adverse Events (NCI CTCAE), version 3.0. History, physical exam, hematology, blood chemistry, and urinalysis were performed at baseline and regular intervals while receiving treatment.

### Evaluation of Efficacy

Treatment efficacy was evaluated by diagnostic imaging per Response Evaluation Criteria in Solid Tumors (RECIST) 1.0 [49]. Radiologic assessments were conducted at baseline and about every 8 weeks thereafter.

### Molecular Testing

EGFR, KRAS, PIK3CA, p53, and PTEN mutation analysis, as well as PTEN expression by immunohistochemistry, were performed in the Clinical Laboratory Improvement Amendments (CLIA)-approved M.D. Anderson Cancer Center laboratory for patients with available archived tissue. For EGFR (exons 18-21 of the kinase domain), KRAS (codons 12, 13 and 61), PIK3CA (codons 532-554 in exon 9 and codons 1011-1062 in exon 20), p53 (exons 4-9), and PTEN mutation (exons 1-9 (entire coding sequence)) testing, PCR-based sequencing analysis was performed on DNA extracted from paraffin-embedded tumor tissue. The lower limit of detection was approximately one cell bearing the mutation per five to ten normal cells. PTEN expression was determined by immunohistochemistry using anti-PTEN monoclonal mouse antibody (Dako, Carpinteria, CA).

### Statistical Analysis

No formal hypotheses were tested, and analyses were descriptive and exploratory. Non-parametric correlations were determined with Spearman's rank correlation coefficient.
